# Unmet Need for Mental Health Services Among High School Students Experiencing Persistent Feelings of Sadness or Hopelessness — Youth Risk Behavior Survey, United States, 2025

**DOI:** 10.15585/mmwr.su7501a4

**Published:** 2026-10-15

**Authors:** Sandra J. Kiplagat, Jorge V. Verlenden, Emiko Petrosky, Simone C. Gray, Adriane King, Jelonia T. Rumph, Marci F. Hertz, J. Michael Underwood

**Affiliations:** ^1^Division of Adolescent and School Health, National Center for Chronic Disease Prevention and Health Promotion, CDC, Atlanta, Georgia; ^2^U.S. Public Health Service, Rockville, Maryland

## Abstract

For the first time, in 2025, CDC’s nationally representative Youth Risk Behavior Survey (YRBS) included an item assessing U.S. high school students’ use of mental health services. Data from this survey were used to examine the prevalence of persistent feelings of sadness or hopelessness among U.S. high school students and their receipt of counseling or therapy. Overall, 33.3% of students reported persistent feelings of sadness or hopelessness, with a higher prevalence among female than male students (43.4% versus 23.9%). Among students reporting persistent feelings of sadness or hopelessness, 29.6% reported receiving counseling or therapy, 28.8% reported an unmet need for counseling or therapy, and 41.5% reported no perceived need for counseling or therapy. A higher prevalence of female students than male students reported receiving counseling or therapy (33.2% versus 23.3%) and an unmet need for counseling or therapy (31.7% versus 23.8%) among those reporting persistent feelings of sadness or hopelessness. Conversely, a higher prevalence of male students than female students reported no perceived need for counseling or therapy (52.9% versus 35.1%). Asian and Black or African American students had both the lowest prevalence of receiving counseling or therapy (15.7% and 15.4%, respectively) and the highest prevalence of an unmet need for counseling or therapy (38.4% and 33.9%, respectively). After controlling for sex, grade, and race and ethnicity, students with persistent feelings of sadness or hopelessness were approximately twice as likely to report receiving counseling or therapy and three times as likely to report an unmet need compared with students who did not report these feelings. These findings highlight potential opportunities for tailored interventions to support adolescent use of mental health services, particularly approaches that address the unmet need for mental health services among female students and the low perceived need for services among male students. Strategies that might improve access to care include school-based mental health services and culturally appropriate family and community engagement.

## Introduction

Adolescence is a period of rapid developmental, emotional, cognitive, and social transition. These changes, combined with increasing academic demands, social pressures, and challenges with identity formation, can increase the risk for mental health disorders among youths (*1*). Data from the 2023 national Youth Risk Behavior Survey (YRBS) indicate that 40% of U.S. high school students reported persistent feelings of sadness or hopelessness, and 29% reported poor mental health, underscoring the magnitude of this public health concern (*2*). Despite the high prevalence of poor mental health, many adolescents do not receive mental health services because of barriers that can be broadly grouped as personal (e.g., stigma), interpersonal (e.g., lack of trusted relationships), and structural factors (e.g., cost, transportation barriers, and limited availability of mental health providers) (*3*,*4*). Although receipt of evidence-based mental health services is associated with improved outcomes (*5*), little is known about the extent to which adolescents experiencing persistent feelings of sadness or hopelessness access counseling or treatment services. Research suggests that mental health symptoms might vary by sex, grade, and race and ethnicity (*2*), yet it remains unclear how these demographic characteristics are associated with access to mental health services among U.S. high school students.

This report uses 2025 YRBS data to provide the first nationally representative estimates of mental health service use among adolescents experiencing persistent feelings of sadness or hopelessness. Previous research has shown a strong association between having felt sad or hopeless for ≥2 weeks in the past year with moderate to severe depressive symptoms, frequent mental distress, and functional limitations resulting from poor mental health (*6*). The goal is to identify critical gaps in the mental health care needs of adolescents, particularly among youths experiencing persistent feelings of sadness or hopelessness, and to guide efforts that promote accessible mental health services for adolescents. Findings from this report are intended for public health practitioners, mental health providers, school and education professionals, researchers, and federal, state, and local mental health and public health agencies.

## Methods

### Data Source

This report includes data from the 2025 YRBS (N = 10,710), a cross-sectional, school-based survey conducted biennially since 1991. Each survey year, CDC collects data from a nationally representative sample of students from public and private schools, including parochial schools, in grades 9–12 in the 50 U.S. states and the District of Columbia. Additional information about YRBS sampling, data collection, response rates, and processing is available in the overview report of this supplement (*7*). The prevalence estimates for use of mental health counseling or therapy and other YRBS variables among U.S. high school students overall and stratified by demographic characteristics are available at https://youthonline.cdc.gov. The full YRBS questionnaire, datasets, and documentation are available at https://www.cdc.gov/yrbs/index.html. This study was approved by institutional review boards at CDC and ICF, the survey contractor, and was conducted consistent with applicable federal law and CDC policy.*

### Measures

Questions, response options, and analytic coding for persistent feelings of sadness or hopelessness and use of mental health counseling or therapy are provided (Table 1). Persistent feelings of sadness or hopelessness were assessed with the question, “During the past 12 months, did you ever feel so sad or hopeless almost every day for two weeks or more in a row that you stopped doing some usual activities?” with “yes” or “no” response options. The mental health counseling or therapy question was administered independently of the persistent feelings of sadness or hopelessness question and did not depend on whether a student responded “yes” to experiencing persistent feelings of sadness or hopelessness. The question assessed counseling or therapy for a broader range of concerns, including emotions, concentration, behavior, or mental health. Students were asked, “During the past 12 months, which of the following best describes your experience with counseling or therapy from a health professional, such as a doctor, nurse, psychologist, or therapist to help with emotions, concentration, behavior, or mental health?” Students selected from the following response options: “I did not receive counseling or therapy because I did not need it” (defined as no perceived need), “I needed counseling or therapy but did not get it because of cost, not knowing how or where to get help, or another reason” (defined as unmet need), and “I received counseling or therapy” (Table 1). Of the 10,710 students in the analytic sample, 1,941 were not asked the mental health counseling or therapy question because of survey procedures for schools selected for both the national and site-specific YRBS samples. Students in these schools completed only one questionnaire; when the site-specific questionnaire was administered, it might not have included all questions on the national YRBS (*7*), including the mental health counseling or therapy question. Among the 8,769 students who were administered the mental health counseling or therapy question, 569 did not provide a valid response, resulting in 8,200 students with valid mental health counseling or therapy data. For the persistent feelings of sadness or hopelessness question, 98 of the 10,710 students in the analytic sample were not asked the question because they completed a site-specific questionnaire that did not include the persistent feelings of sadness or hopelessness question. Among the 10,612 students who were administered the persistent feelings of sadness or hopelessness question, 129 did not provide a valid response, resulting in 10,483 students with valid data on persistent feelings of sadness or hopelessness. A total of 8,049 students had valid responses to both the persistent feelings of sadness or hopelessness and the mental health counseling or therapy questions.

**TABLE 1:** Questions, response options, and analytic coding for persistent feelings of sadness or hopelessness and use of mental health services — Youth Risk Behavior Survey, United States, 2025

Variable	Question	Response option	Analytic coding
Persistent feelings of sadness or hopelessness	During the past 12 months, did you ever feel so sad or hopeless almost every day for two weeks or more in a row that you stopped doing some usual activities?	Yes or no	Yes versus no
Use of mental health counseling or therapy	During the past 12 months, which of the following best describes your experience with counseling or therapy from a health professional, such as a doctor, nurse, psychologist, or therapist to help with emotions, concentration, behavior, or mental health?	I did not receive counseling or therapy because I did not need it; I needed counseling or therapy but did not get it because of cost, not knowing how or where to get help, or another reason; I received counseling or therapy	Did not receive counseling or therapy because I did not need it (no perceived need) versus unmet need for counseling or therapy (unmet need) versus received counseling or therapy

Demographic variables included sex (female or male), grade (9, 10, 11, or 12), and race and ethnicity (American Indian or Alaska Native [AI/AN], Asian, Black or African American [Black], Hispanic or Latino [Hispanic], Middle Eastern or North African [ME/NA], Native Hawaiian or Pacific Islander [NH/PI], White, or multiracial [selected more than one racial category]). Persons of Hispanic origin might be of any race but are categorized as Hispanic; all racial groups are non-Hispanic.

## Analysis

Weighted prevalence estimates and 95% CIs were calculated for persistent feelings of sadness or hopelessness and receipt of, unmet need for, and no perceived need for mental health counseling or therapy. Among students who reported persistent feelings of sadness or hopelessness, prevalence and 95% CIs were estimated for all three counseling or therapy outcomes. Differences in prevalence by demographic characteristics were assessed using design-adjusted *t* tests for pairwise comparisons. Corrections for multiple comparisons were not applied because the analyses were descriptive and hypothesis generating, and each statistical test addressed a distinct research question and was interpreted independently. Taylor series linearization was used for all estimates and measures of association, with statistical significance set at p<0.05. Prevalence estimates with a denominator <30 were considered statistically unreliable and were suppressed (*7*).

Adjusted prevalence ratios (aPRs) and 95% CIs for the association between persistent feelings of sadness or hopelessness and mental health counseling or therapy outcomes were calculated using logistic regression models with predicted marginals. Sex-stratified analyses were conducted a priori to assess whether associations between persistent feelings of sadness or hopelessness and mental health counseling or therapy differed between female and male adolescents, given previously documented sex differences in adolescent mental health symptoms and help-seeking behaviors (*2*). Overall models controlled for sex, grade, and race and ethnicity; sex-stratified models controlled for grade and race and ethnicity. Prevalence ratios were considered statistically significant if the 95% CI excluded the null hypothesis value of 1.0. All analyses were conducted using SAS-callable SUDAAN (version 11.0.4; RTI International) to account for the complex sampling design and weighting.

## Results

Overall, in 2025, 33.3% of U.S. high school students reported persistent feelings of sadness or hopelessness. The prevalence was higher among female students than male students (43.4% versus 23.9%) and similar across grade levels (Table 2). Across racial and ethnic groups, the highest prevalence was among NH/PI students (40.6%), followed by AI/AN (39.5%) and multiracial students (39.3%); prevalence was lowest among ME/NA students (20.9%).

**TABLE 2:** Prevalence of persistent feelings of sadness or hopelessness among high school students, by demographic characteristics — Youth Risk Behavior Survey, United States, 2025*

Characteristic	Persistent feelings of sadness or hopelessness during the past 12 months
	% (95% CI)
**Overall**	**33.3 (31.2–35.5)**
**Sex**	
Female^†^	43.4 (40.1–46.8)
Male	23.9 (21.9–26.0)
**Grade**	
9	32.7 (29.9–35.7)
10	34.2 (31.4–37.1)
11	34.3 (30.8–38.0)
12	32.0 (29.1–35.1)
**Race and ethnicity^§^**	
AI/AN^¶^	39.5 (27.2–53.2)
Asian**	23.9 (18.7–29.9)
Black or African American^††^	34.3 (31.7–37.0)
Hispanic or Latino^§§^	37.0 (34.1–40.0)
ME/NA^¶¶^	20.9 (11.7–34.5)
NH/PI***	40.6 (33.2–48.6)
White	31.5 (28.1–35.1)
Multiracial^†††^	39.3 (33.7–45.2)

**Abbreviations:** AI/AN = American Indian or Alaska Native; ME/NA = Middle Eastern or North African; NH/PI = Native Hawaiian or Pacific Islander.

* N = 10,483 respondents with valid responses for the persistent feelings of sadness or hopelessness question (of 10,710 participants in the 2025 national YRBS). The total number (N) of students answering each question varied. Data might be missing because 1) students in schools selected for both national and site-specific YRBS samples completed only one questionnaire; therefore, when the site-specific questionnaire was administered, it might not have included all questions on the national YRBS; 2) the student did not answer the question; or 3) the response was set to missing because of an out-of-range response or logical inconsistency. Prevalence estimates in each category are calculated based on nonmissing data.

^†^ Female students differed significantly from male students in the prevalence of persistent feelings of sadness or hopelessness as determined by *t* test analysis with Taylor series linearization (p<0.05).

^§^ Persons of Hispanic or Latino (Hispanic) origin might be of any race but are categorized as Hispanic; all racial groups are non-Hispanic.

^¶^ AI/AN students differed significantly from Asian and ME/NA students in the prevalence of persistent feelings of sadness or hopelessness as determined by *t* test analysis with Taylor series linearization (p<0.05).

** Asian students differed significantly from Black or African American (Black), Hispanic, NH/PI, multiracial, and White students in the prevalence of persistent feelings of sadness or hopelessness as determined by *t* test analysis with Taylor series linearization (p<0.05).

^††^ Black students differed significantly from ME/NA students in the prevalence of persistent feelings of sadness or hopelessness as determined by *t* test analysis with Taylor series linearization (p<0.05).

^§§^ Hispanic students differed significantly from ME/NA and White students in the prevalence of persistent feelings of sadness or hopelessness as determined by *t* test analysis with Taylor series linearization (p<0.05).

^¶¶^ ME/NA students differed significantly from NH/PI and multiracial students in the prevalence of persistent feelings of sadness or hopelessness as determined by *t* test analysis with Taylor series linearization (p<0.05).

*** NH/PI students differed significantly from White students in the prevalence of persistent feelings of sadness or hopelessness as determined by *t* test analysis with Taylor series linearization (p<0.05).

^†††^ Multiracial students differed significantly from White students in the prevalence of persistent feelings of sadness or hopelessness as determined by *t* test analysis with Taylor series linearization (p<0.05).

Overall, 18.6% of students reported receiving counseling or therapy, 15.4% reported an unmet need for counseling or therapy, and 66.0% reported no perceived need for counseling or therapy (Table 3). Compared with male students, female students had a higher prevalence of receiving counseling or therapy (25.1% versus 12.3%) and reporting an unmet need for counseling or therapy (21.1% versus 9.9%). Conversely, a higher prevalence of male students than female students reported no perceived need for counseling or therapy (77.7% versus 53.7%). Compared with other racial and ethnic groups, Asian students had the lowest prevalence of receiving counseling or therapy (6.4%) and the highest prevalence of reporting no perceived need for counseling or therapy (77.4%).

**TABLE 3:** Use of mental health services among high school students, by demographic characteristics — Youth Risk Behavior Survey, United States, 2025*

Characteristic	Received counseling or therapy	Unmet need for counseling or therapy	No perceived need for counseling or therapy
	% (95% CI)	% (95% CI)	% (95% CI)
**Overall**	**18.6 (16.5–20.9)**	**15.4 (14.5–16.4)**	**66.0 (63.8–68.1)**
**Sex**			
Female^†^	25.1 (22.2–28.3)	21.1 (19.4–23.0)	53.7 (50.8–56.6)
Male	12.3 (10.9–13.9)	9.9 (8.8–11.1)	77.7 (76.1–79.3)
**Grade**			
9	16.3 (13.5–19.4)^§^	14.7 (13.0–16.6)	69.0 (66.1–71.8)^¶^
10	20.2 (17.1–23.6)	15.3 (13.1–17.7)	64.6 (60.7–68.2)
11	18.4 (15.6–21.4)	17.2 (14.8–19.9)	64.4 (60.9–67.8)
12	19.8 (16.4–23.8)	14.7 (12.3–17.5)	65.5 (61.7–69.1)
Race and ethnicity**^,††^			
AI/AN	8.1 (3.3–18.3)^§§^	19.1 (8.2–38.7)	72.8 (55.2–85.3)
Asian	6.4 (4.0–10.0)^¶¶^	16.2 (13.2–19.7)	77.4 (73.2–81.1)***
Black or African American	12.8 (9.9–16.4)^†††^	17.2 (14.8–19.8)	70.0 (65.8–74.0)^§§§^
Hispanic or Latino	16.8 (14.4–19.5)^¶¶¶^	15.9 (13.8–18.2)	67.3 (63.9–70.6)****
ME/NA	20.2 (10.8–34.7)	13.3 (7.0–23.9)	66.4 (53.7–77.1)
White	21.6 (19.2–24.3)	14.7 (13.1–16.4)	63.6 (60.7–66.5)
Multiracial	24.8 (20.6–29.6)	15.6 (12.3–19.6)	59.5 (54.2–64.7)

**Abbreviations:** AI/AN = American Indian or Alaska Native; ME/NA = Middle Eastern or North African.

* N = 8,200 respondents with valid responses for the mental health service use item (of 10,710 participants in the 2025 national YRBS). The total number (N) of students answering each question varied. Data might be missing because 1) students in schools selected for both national and site-specific YRBS samples completed only one questionnaire; therefore, when the site-specific questionnaire was administered, it might not have included all questions on the national YRBS; 2) the student did not answer the question; or 3) the response was set to missing because of an out-of-range response or logical inconsistency. Prevalence estimates in each category are calculated based on nonmissing data.

^†^ Female students differed significantly from male students in the prevalence of receiving counseling or therapy, unmet need, and no perceived need as determined by *t* test analysis with Taylor series linearization (p<0.05).

^§^ Students in grade 9 differed significantly from students in grades 10 and 12 in the prevalence of receiving counseling or therapy as determined by *t* test analysis with Taylor series linearization (p<0.05).

^¶^ Students in grade 9 differed significantly from students in grades 10 and 11 in the prevalence of no perceived need as determined by *t* test analysis with Taylor series linearization (p<0.05).

** Persons of Hispanic or Latino (Hispanic) origin might be of any race but are categorized as Hispanic; all racial groups are non-Hispanic.

^††^ Prevalence estimates with a denominator <30 were considered statistically unreliable and were suppressed; therefore, estimates for Native Hawaiian or Pacific Islander students are not presented.

^§§^ AI/AN students differed significantly from Hispanic, multiracial, and White students in the prevalence of receiving counseling or therapy as determined by *t* test analysis with Taylor series linearization (p<0.05).

^¶¶^ Asian students differed significantly from Black or African American (Black), Hispanic, ME/NA, multiracial, and White students in the prevalence of receiving counseling or therapy as determined by *t* test analysis with Taylor series linearization (p<0.05).

*** Asian students differed significantly from Black, Hispanic, multiracial, and White students in the prevalence of no perceived need for counseling or therapy as determined by *t* test analysis with Taylor series linearization (p<0.05).

^†††^ Black students differed significantly from Hispanic, multiracial, and White students in the prevalence of receiving counseling or therapy as determined by *t* test analysis with Taylor series linearization (p<0.05).

^§§§^ Black students differed significantly from multiracial and White students in the prevalence of no perceived need for counseling or therapy as determined by *t* test analysis with Taylor series linearization (p<0.05).

^¶¶¶^ Hispanic students differed significantly from multiracial and White students in the prevalence of receiving counseling or therapy as determined by *t* test analysis with Taylor series linearization (p<0.05).

**** Hispanic students significantly differed from multiracial students in the prevalence of no perceived need for counseling or therapy as determined by *t* test analysis with Taylor series linearization (p<0.05).

Among students reporting persistent feelings of sadness or hopelessness, 29.6% reported receiving counseling or therapy, 28.8% reported an unmet need for counseling or therapy, and 41.5% reported no perceived need for counseling or therapy (Table 4). Compared with male students, female students had a higher prevalence of receiving counseling or therapy (33.2% versus 23.3%) and reporting an unmet need for counseling or therapy (31.7% versus 23.8%). Conversely, a higher prevalence of male students than female students reported no perceived need for counseling or therapy (52.9% versus 35.1%).

**TABLE 4:** Mental health service use among students who reported persistent feelings of sadness or hopelessness for ≥2 weeks during the past 12 months, by demographic characteristics — Youth Risk Behavior Survey, United States, 2025*

Characteristic	Received counseling or therapy	Unmet need for counseling or therapy	No perceived need for counseling or therapy
	% (95% CI)	% (95% CI)	% (95% CI)
**Overall**	**29.6 (25.4–34.2)**	**28.8 (26.8–31.0)**	**41.5 (38.0–45.2)**
**Sex**			
Female^†^	33.2 (28.5–38.4)	31.7 (29.1–34.3)	35.1 (31.0–39.3)
Male	23.3 (18.8–28.5)	23.8 (20.1–27.8)	52.9 (48.6–57.2)
**Grade**			
9	26.6 (21.5–32.4)	29.6 (26.6–32.9)	43.8 (38.8–48.9)
10	31.7 (25.7–38.4)	28.9 (25.5–32.5)	39.4 (33.5–45.6)
11	28.1 (22.4–34.5)	29.4 (24.6–34.7)	42.5 (36.7–48.5)
12	32.5 (26.3–39.4)	27.3 (22.9–32.2)	40.2 (33.8–46.8)
**Race and ethnicity^§,¶^**			
Asian	15.7 (9.0–26.1)**	38.4 (30.1–47.5)^††^	45.8 (37.2–54.7)^§§^
Black or African American	15.4 (11.0–21.1)^¶¶^	33.9 (29.0–39.0)***	50.8 (43.6–57.9)^†††^
Hispanic or Latino	25.1 (20.6–30.1)^§§§^	29.0 (25.1–33.2)	46.0 (40.9–51.1)^¶¶¶^
White	36.5 (31.8–41.5)	27.1 (24.6–29.9)	36.3 (32.6–40.2)
Multiracial	39.6 (30.5–49.4)	24.4 (18.5–31.4)	36.0 (29.9–42.6)

* N = 3,623 respondents who responded yes to the item assessing persistent feelings of sadness or hopelessness for ≥2 weeks during the past 12 months (of 10,710 participants in the 2025 national YRBS). The total number (N) of students answering each question varied. Data might be missing because 1) students in schools selected for both national and site-specific YRBS samples completed only one questionnaire; therefore, when the site-specific questionnaire was administered, it might not have included all questions on the national YRBS; 2) the student did not answer the question; or 3) the response was set to missing because of an out-of-range response or logical inconsistency. Prevalence estimates in each category are calculated based on nonmissing data.

^†^ Among students who reported persistent feelings of sadness or hopelessness, female students differed significantly from male students in the prevalence of receiving counseling or therapy, unmet need, and no perceived need as determined by *t* test analysis with Taylor series linearization (p<0.05).

^§^ Persons of Hispanic or Latino (Hispanic) origin might be of any race but are categorized as Hispanic; all racial groups are non-Hispanic.

^¶^ Prevalence estimates with a denominator <30 were considered statistically unreliable and were suppressed; therefore, estimates for American Indian or Alaska Native, Middle Eastern or North African, and Native Hawaiian or Pacific Islander students are not presented.

** Among students who reported persistent feelings of sadness or hopelessness, Asian students significantly differed from Hispanic, multiracial, and White students in the prevalence of receiving counseling or therapy as determined by *t* test analysis with Taylor series linearization (p<0.05).

^††^ Among students who reported persistent feelings of sadness or hopelessness, Asian students significantly differed from Hispanic, multiracial, and White students in the prevalence of unmet need for counseling or therapy as determined by *t* test analysis with Taylor series linearization (p<0.05).

^§§^ Among students who reported persistent feelings of sadness or hopelessness, Asian students significantly differed from White students in the prevalence of no perceived need for counseling or therapy as determined by *t* test analysis with Taylor series linearization (p<0.05).

^¶¶^ Among students who reported persistent feelings of sadness or hopelessness, Black or African American (Black) students significantly differed from Hispanic, multiracial, and White students in the prevalence of receiving counseling or therapy as determined by *t* test analysis with Taylor series linearization (p<0.05).

*** Among students who reported persistent feelings of sadness or hopelessness, Black students significantly differed from multiracial and White students in the prevalence of unmet need for counseling or therapy as determined by *t* test analysis with Taylor series linearization (p<0.05).

^†††^ Among students who reported persistent feelings of sadness or hopelessness, Black students significantly differed from multiracial and White students in the prevalence of no perceived need for counseling or therapy as determined by *t* test analysis with Taylor series linearization (p<0.05).

^§§§^ Among students who reported persistent feelings of sadness or hopelessness, Hispanic students significantly differed from multiracial and White students in the prevalence of receiving counseling or therapy as determined by *t* test analysis with Taylor series linearization (p<0.05).

^¶¶¶^ Among students who reported persistent feelings of sadness or hopelessness, Hispanic students significantly differed from multiracial and White students in the prevalence of no perceived need for counseling or therapy as determined by *t* test analysis with Taylor series linearization (p<0.05).

Differences in receipt of counseling or therapy were observed by race and ethnicity. Among those reporting persistent feelings of sadness or hopelessness, receipt of counseling or therapy was lowest among Black (15.4%) and Asian (15.7%) students and highest among multiracial (39.6%) and White (36.5%) students (Table 4). Unmet need for counseling or therapy was highest among Asian (38.4%) and Black (33.9%) students. Compared with other racial and ethnic groups, the prevalence of reporting no perceived need for counseling or therapy was highest among Black (50.8%), Hispanic (46.0%), and Asian students (45.8%).

After adjusting for sex, grade, and race and ethnicity, students reporting persistent feelings of sadness or hopelessness were more likely to report receiving counseling or therapy (aPR = 2.08; 95% CI = 1.83–2.36) and more likely to report an unmet need (aPR = 3.05; 95% CI = 2.62–3.56) compared with students who did not report these feelings (Table 5). Conversely, students reporting persistent feelings of sadness or hopelessness were less likely to report no perceived need for counseling or therapy (aPR = 0.57; 95% CI = 0.54–0.61). In sex-stratified analyses, findings were consistent with the overall adjusted model for both male and female students.

**TABLE 5:** Association between persistent feelings of sadness or hopelessness and mental health service use, by sex — Youth Risk Behavior Survey, United States, 2025

Mental health service use	Persistent feelings of sadness or hopelessness								
	Overall			Female			Male		
	Yes	No	aPR*	Yes	No	aPR^†^	Yes	No	aPR^†^
	% (95% CI)	% (95% CI)	% (95% CI)	% (95% CI)	% (95% CI)	% (95% CI)	% (95% CI)	% (95% CI)	% (95% CI)
Received counseling or therapy	**29.6 (25.4–34.2)**	**12.8 (11.2–14.7)**	**2.08 (1.83–2.36)**	33.2 (28.5–38.4)	18.5 (15.9–21.3)	1.83 (1.59–2.11)	23.3 (18.8–28.5)	8.9 (7.6–10.2)	2.65 (2.09–3.36)
Unmet need for counseling or therapy	**28.8 (26.8–31.0)**	**8.5 (7.6–9.6)**	**3.05** **(2.62–3.56)**	31.7 (29.2–34.3)	12.8 (11.3–14.5)	2.52 (2.21–2.87)	23.8 (20.1–27.8)	5.5 (4.4–6.7)	4.20 (3.11–5.69)
No perceived need for counseling or therapy	**41.5 (38.0–45.2)**	**78.6 (76.4–80.7)**	**0.57 (0.54–0.61)**	35.1 (31.0–39.3)	68.7 (65.6–71.7)	0.50 (0.45–0.55)	52.9 (48.6–57.2)	85.7 (83.9–87.3)	0.62 (0.57–0.68)

**Abbreviation:** aPR = adjusted prevalence ratio.

* Adjusted for sex, grade, and race and ethnicity. The aPR compares students with persistent feelings of sadness or hopelessness (answered “yes”) to those without (answered “no”; referent group).

^†^ Adjusted for grade and race and ethnicity. The aPR compares students with persistent feelings of sadness or hopelessness (answered “yes”) to those without (answered “no”; referent group).

## Discussion

This report provides the first nationally representative estimates for receipt of, unmet need for, and no perceived need for counseling or therapy among U.S. high school students reporting persistent feelings of sadness or hopelessness, a key measure collected by YRBS that has been associated with depressive symptoms (*6*). In 2025, approximately one in three students reported persistent feelings of sadness or hopelessness. Of those students, only about one third reported receiving counseling or therapy. An additional one third reported an unmet need for counseling or therapy, indicating they wanted help but did not get it because of cost, not knowing how or where to get help, or another reason. These findings are consistent with household-based surveys, such as the 2024 National Survey on Drug Use and Health, which found that among adolescents aged 12–17 years with a past-year major depressive episode who did not receive mental health treatment, 34.2% reported not seeking treatment despite believing they needed it (*8*).

Patterns of mental health service use and perceived need for services varied significantly by sex and race and ethnicity. Consistent with previous research, the prevalence of persistent feelings of sadness or hopelessness among female students was nearly twice that of male students (*2*). Pressures to conform to gender-role expectations and hormonal changes associated with puberty have been proposed as potential contributing factors to the emergence of sex differences in adolescent depression (*9*). During puberty, biologic changes occur alongside psychosocial transitions that might interact in ways associated with greater risk for depressive symptoms, particularly among girls (*9*). Although female students had a higher prevalence of receiving counseling or therapy than male students, they also had a higher prevalence of unmet need for counseling or therapy. Conversely, male students had a higher prevalence of reporting no perceived need for counseling or therapy. For male students, lower perceived need and lower use of mental health services might be related to social norms that stigmatize poor mental health and seeking help as signs of personal weakness (*10*). Although seeking help for mental health concerns might be considered more acceptable for females, structural and interpersonal barriers such as the cost of services, limited provider availability, and negative family attitudes might impede access for students, regardless of sex (*3*,*4*).

Differences were also apparent by race and ethnicity. Among students reporting persistent feelings of sadness or hopelessness, Black and Asian students had the lowest prevalence of receiving counseling or therapy and the highest prevalence of unmet need; in addition, Black, Asian, and Hispanic students had the highest prevalence of reporting no perceived need for counseling or therapy. Although these data do not allow for a direct examination of why these students did not receive counseling or therapy or perceived that they did not need care, previous research suggests that stigma, parental beliefs, and structural barriers such as low income and insurance status might discourage both acknowledgment of need and formal help-seeking behaviors among racial and ethnic minorities (*11*). For all students, multiple barriers might impede access to mental health services. Frequently cited structural barriers include financial constraints, transportation issues, long wait times, and difficulty navigating health resources (*3*,*4*,*8*). Personal and interpersonal factors, such as stigma, low perceived symptom severity, and a preference for self-reliance, also might discourage youths from seeking help (*3*,*4*,*8*). Negative family and community attitudes toward mental health concerns, including distrust of health professionals or skepticism regarding treatment effectiveness, have been associated with lower help-seeking behaviors and use of mental health services (*3*). Because past negative experiences with health care providers have been associated with low trust in mental health services (*3*,*12*), creating supportive, adolescent-friendly care environments might help reduce barriers to mental health service use.

Population-level strategies, such as improving mental health literacy, might help address these multifaceted challenges. Mental health literacy is the ability to recognize warning signs of poor mental health, understand when and how to seek help from trusted adults or mental health professionals, and use healthy coping strategies to manage stress and promote well-being (*3*). Health education, generally, and efforts to increase mental health literacy, specifically, have been associated with increased use of health care and positive engagement with health care providers (*3*).

Schools might play an important role in promoting mental health and supporting students facing a wide range of emotional and mental health concerns (*13*). For example, school-based mental health services might improve access by reducing transportation and cost barriers for families. In 2025, 55% of public school administrators reported an increase in the percentage of school staff members expressing concerns about students exhibiting depression, anxiety, trauma, or emotional dysregulation or disturbance (https://nces.ed.gov/surveys/spp/results.asp). However, only 52% of administrators reported that their school was able to provide mental health services for all students who needed them. Addressing mental health workforce shortages and expanding the school-based mental health workforce might improve access to both prevention and treatment services. Initiatives that streamline referral processes and expand school-based care might help address gaps between the prevalence of mental health challenges and the use of services (*14*).

In addition to providing access to services, schools might play a preventive role by fostering safe and supportive environments that promote mental health and well-being. In 2023, CDC released Promoting Mental Health and Well-Being in Schools: An Action Guide for School and District Leaders (https://www.cdc.gov/mental-health-action-guide/about/index.html), which outlines evidence-based strategies to support student mental health and well-being. Key strategies include increasing students’ mental health literacy by delivering classroom-based mental health education and implementing peer modeling programs that support students at all grade levels to build resilience and manage emotional challenges. CDC–supported programs in several school districts have implemented evidence-based approaches incorporating trauma-informed practices, mindfulness activities, and social-emotional skill development to support student mental health and well-being, consistent with recommended school-based mental health promotion strategies. For example, Chicago Public Schools adopted a comprehensive mental health and suicide prevention policy in 2022 (https://www.cps.edu/sites/cps-policy-rules/policies/700/704/704-13/) that requires each of its 647 K–12 schools to establish a behavioral health team responsible for providing universal and targeted prevention and composed of a school psychologist, nurses, case managers, social workers, and other relevant stakeholders. Other CDC–supported recipients, such as the Arkansas Department of Education and Eaton Regional Education Services Agency, have leveraged CDC’s What Works in Schools program (https://www.cdc.gov/healthy-youth/what-works-in-schools/overview.html) and the Substance Abuse and Mental Health Services Administration’s (SAMHSA’s) Project AWARE (Advancing Wellness and Resiliency in Education) program (https://www.samhsa.gov/mental-health/children-and-families/school-health/project-aware) to support sustainable infrastructure for school-based mental health programs and services, including primary prevention.

Programs like these provide examples of how schools can move beyond service provision alone to proactively support students’ emotional development and overall well-being in a classroom setting through implementation of comprehensive, multitiered systems of support and collaboration with community-based partners. Integrating mental health services into primary care, expanding telehealth capacity, and increasing school-based counseling services might improve the continuity of care for adolescents (*14*). Strengthening trusting relationships between youths and parents, teachers, and counselors might facilitate help-seeking behaviors (*14*). Peer support programs and interventions that equip parents and guardians to recognize signs of distress and support adolescents in seeking help might further reduce barriers to accessing mental health services (*15*). For adolescents and families who are seeking services, care navigator programs might provide additional support by helping them connect with appropriate mental health resources, potentially improving continuity of care and reducing unmet need (*16*).

## Limitations

General limitations for YRBS are available in the overview report of this supplement (*7*). The findings in this report are subject to at least three additional limitations. First, the YRBS measure of mental health counseling or therapy does not capture the type, frequency, or quality of services received, nor can it be used to assess the adequacy of care. Second, the survey’s response options for unmet need (e.g., cost, not knowing how or where to get help, or another reason) do not collect specific reasons, which limits the ability to fully examine specific barriers to accessing care. Finally, the majority of missing data for the mental health counseling or therapy item were from students who were not asked the question to reduce respondent effort in schools selected for both national and site-level YRBS samples, which might limit generalizability. However, students with and without mental health counseling or therapy data did not differ significantly by sex, race and ethnicity, or grade.

## Future Directions

Given the higher prevalence of unmet need for mental health counseling or therapy among female and racial and ethnic minority students experiencing persistent feelings of sadness or hopelessness, future research is needed to identify effective strategies for addressing barriers to care in these populations. Although evidence-based primary prevention strategies have been identified, additional research is needed to evaluate their implementation and effectiveness in schools and community settings, because improving access to clinical services alone might not fully address adolescent mental health needs. Potential primary prevention strategies include mental health education, violence prevention programming, peer mentoring, brief interventions, support groups, and other low-intensity, school-based supports.

Studies should also explore how adolescents access mental health information online, including through social media and generative artificial intelligence (AI). Approximately one in eight U.S. adolescents reported using generative AI chatbots for mental health support (*17*). Although these technologies might reduce barriers related to cost, accessibility, and stigma, concerns remain regarding the lack of scientific validation, safety protocols, and regulatory oversight (https://www.apa.org/topics/artificial-intelligence-machine-learning/health-advisory-ai-chatbots-wellness-apps-mental-health.pdf). Potential priorities include developmentally appropriate digital literacy education, dissemination of accurate mental health information, and support for parents, caregivers, and school professionals in guiding adolescents’ use of these technologies.

## Conclusion

Approximately one in three U.S. high school students reported persistent feelings of sadness or hopelessness, yet fewer than one third of these students received counseling or therapy. An unmet need for mental health counseling or therapy was most prevalent among female students and certain racial and ethnic groups, whereas male students had a higher prevalence of reporting no perceived need for counseling or therapy. These findings highlight potential opportunities for a multitiered response that includes school-based mental health services, improved mental health literacy, and socially and culturally appropriate interventions that engage all youths, families, and communities. CDC resources such as Promoting Mental Health and Well-Being in Schools: An Action Guide for School and District Leaders, as well as What Works in Schools, might support implementation of such approaches in school settings. Increasing access to appropriate and inclusive mental health support for adolescents remains a public health priority.

## References

[1] Suppiej A, Longo I, Pettoello-Mantovani M. The pivotal role of mental health in child and adolescent development. Glob Pediatr 2025;13:100277. 10.1016/j.gpeds.2025.100277

[2] Verlenden JV, Fodeman A, Wilkins N, Mental health and suicide risk among high school students and protective factors—Youth Risk Behavior Survey, United States, 2023. In: Youth Risk Behavior Surveillance—United States, 2023. MMWR Suppl 2024;73(No. Suppl 4):79–86. 10.15585/mmwr.su7304a9 39378246 PMC11559681

[3] Aguirre Velasco A, Cruz ISS, Billings J, Jimenez M, Rowe S. What are the barriers, facilitators and interventions targeting help-seeking behaviours for common mental health problems in adolescents? A systematic review. BMC Psychiatry 2020;20:293. 10.1186/s12888-020-02659-0 32527236 PMC7291482

[4] Gulliver A, Griffiths KM, Christensen H. Perceived barriers and facilitators to mental health help-seeking in young people: a systematic review. BMC Psychiatry 2010;10:113. 10.1186/1471-244x-10-113 21192795 PMC3022639

[5] Das JK, Salam RA, Lassi ZS, Interventions for adolescent mental health: an overview of systematic reviews. J Adolesc Health 2016;59(Suppl):S49–60. 10.1016/j.jadohealth.2016.06.020 27664596 PMC5026677

[6] Verlenden J, Pampati S, Heim Viox M, Measuring population-level adolescent mental health using a single-item indicator of experiences of sadness and hopelessness: cross-sectional study. JMIR Form Res 2024;8:e54288. 10.2196/54288 39059010 PMC11316145

[7] Mpofu JJ, McKinnon II, Figueroa A, Overview and methods for the Youth Risk Behavior Surveillance System—United States, 2025. In: Youth Risk Behavior Surveillance—United States, 2025. MMWR Suppl 2026;75(No. Suppl 1):1–11. 10.15585/mmwr.su7501a1 PMC13660954 42868098

[8] Substance Abuse and Mental Health Services Administration. Key substance use and mental health indicators in the United States: results from the 2024 national survey on drug use and health (HHS Publication No. PEP25–07–007, NSDUH Series H-60). Rockville, MD: US Department of Health and Human Services, Substance Abuse and Mental Health Services Administration; 2025. https://library.samhsa.gov/product/2024-nsduh-report/pep25-07-007

[9] Hyde JS, Mezulis AH, Abramson LY. The ABCs of depression: integrating affective, biological, and cognitive models to explain the emergence of the gender difference in depression. Psychol Rev 2008;115:291–313. 10.1037/0033-295X.115.2.291 18426291

[10] Sheikh A, Payne-Cook C, Lisk S, Carter B, Brown JSL. Why do young men not seek help for affective mental health issues? A systematic review of perceived barriers and facilitators among adolescent boys and young men. Eur Child Adolesc Psychiatry 2025;34:565–83. 10.1007/s00787-024-02520-9 39004687 PMC11868194

[11] Lu W, Todhunter-Reid A, Mitsdarffer ML, Muñoz-Laboy M, Yoon AS, Xu L. Barriers and facilitators for mental health service use among racial/ethnic minority adolescents: a systematic review of literature. Front Public Health 2021;9:641605. 10.3389/fpubh.2021.641605 33763401 PMC7982679

[12] Lawrence SM, Saab MM, Savage E, Hegarty J, FitzGerald S. Adolescents’ perspectives and experiences of accessing general practitioner services: a systematic review. J Child Health Care 2025;29:734–54. 10.1177/13674935241239837 38629615 PMC12368295

[13] Fazel M, Hoagwood K, Stephan S, Ford T. Mental health interventions in schools 1: mental health interventions in schools in high-income countries. Lancet Psychiatry 2014;1:377–87. 10.1016/S2215-0366(14)70312-8 26114092 PMC4477835

[14] Woolf SH. The youth mental health crisis in the United States: epidemiology, contributors, and potential solutions. Pediatrics 2025;156:e2025070849. 10.1542/peds.2025-070849 41145167

[15] Choi KR, Easterlin MC. Intervention models for increasing access to behavioral health services among youth: a systematic review. J Dev Behav Pediatr 2018;39:754–62. 10.1097/dbp.0000000000000623 30334855

[16] Petts RA, McClain MB, Azad G, Shahidullah JD. System navigation models to facilitate engagement in pediatric behavioral health services: a systematic review. Fam Syst Health 2021;39:618–31. https://psycnet.apa.org/doi/10.1037/fsh0000643 34618517 10.1037/fsh0000643

[17] McBain RK, Bozick R, Diliberti M, Use of generative AI for mental health advice among US adolescents and young adults. JAMA Netw Open 2025;8:e2542281. 10.1001/jamanetworkopen.2025.42281 41201806 PMC12595529

